# Cardiac Conduction System in Congenitally Corrected Transposition of the Great Arteries and Its Clinical Relevance

**DOI:** 10.1161/JAHA.117.007759

**Published:** 2017-12-21

**Authors:** Alban‐Elouen Baruteau, Dominic J. Abrams, Siew Yen Ho, Jean‐Benoit Thambo, Christopher J. McLeod, Maully J. Shah

**Affiliations:** ^1^ Department of Congenital Cardiology Evelina London Children's Hospital Guy's and St Thomas' NHS Foundation Trust London United Kingdom; ^2^ M3C CHU de Nantes Fédération des Cardiopathies Congénitales Nantes France; ^3^ Department of Cardiology Boston Children's Hospital Harvard Medical School Boston MA; ^4^ Cardiac Morphology Royal Brompton Hospital and Harefield NHS Foundation Trust Imperial College London London United Kingdom; ^5^ Department of Paediatric Cardiology CHU Bordeaux Pessac France; ^6^ IHU LIRYC, Electrophysiology and Heart Modeling Institute Bordeaux France; ^7^ Department of Cardiovascular Diseases Mayo Clinic College of Medicine Mayo Clinic Rochester MN; ^8^ Division of Cardiology Department of Pediatrics Children's Hospital of Philadelphia University of Pennsylvania Philadelphia PA

**Keywords:** congenital heart disease, heart anatomy, heart block, pacing, pediatric, Arrhythmias, Electrophysiology, Mechanisms, Congenital Heart Disease

## Introduction

Congenitally corrected transposition of the great arteries (ccTGA) is a rare cardiac malformation characterized by the combination of discordant atrioventricular and ventriculoarterial connections.^1^ The incidence has been reported to be around 1/33 000 live births, accounting for ≈0.05% of congenital heart malformations.^2^ Although a familial recurrence of heart defects in subjects with ccTGA has been reported,^3^ the etiology of this malformation is not currently known.^1^, ^4^

The morphological right atrium is connected to the morphological left ventricle across the mitral valve, with the left ventricle then connected to the pulmonary trunk; the morphological left atrium is connected to the morphological right ventricle across the tricuspid valve, with the morphological right ventricle connected to the aorta. Because of this “double discordance,” the systemic venous return is pumped to the lungs, while the pulmonary venous return is directed to the body. In the context of usual atrial arrangement (situs solitus), the morphological left ventricle is thus positioned to the right and the aorta, arising from the morphological right ventricle, is more commonly left‐sided. In mirror‐image atrial arrangement (situs inversus), the morphological left ventricle is left‐sided, and the aorta is usually positioned to the right.

Most patients have at least 1 associated cardiac anomaly, mainly ventricular septal defect (VSD), subvalvular and/or valvular pulmonary stenosis, or abnormalities of the systemic (tricuspid) atrioventricular (AV) valve with coexisting accessory pathways.^4^ Because of the unusual position of the atrioventricular node and course of the atrioventricular conduction bundle, cardiac conduction disorders are frequent and markedly alter the natural history and management of the malformation.^5^, ^6^, ^7^

This article provides a comprehensive, up‐to‐date description of the characteristics of the cardiac conduction system in patients with ccTGA, their clinical sequelae, and their contemporary management.

## Morphology and Cardiac Conduction System in ccTGA

Most commonly, there is the usual atrial arrangement. The heart position in the chest is usually normal or midline or occasionally right‐sided. The morphologic right ventricle is often to the left and slightly anterior position or is directly to the left of the morphologic left ventricle in the setting of a vertically oriented septum. Compared with normal hearts, the morphologic right atrium may bear features relevant to interventions such as a displaced coronary sinus orifice, a short isthmus, and a short posterior rim of the oval fossa.^8^

**Figure 1:**
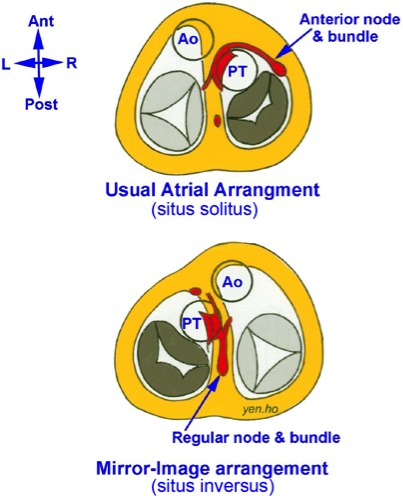
Disposition of the atrioventricular conduction system in ccTGA in usual atrial arrangement (situs solitus, top panel) or in mirror‐image arrangement (situs inversus, bottom panel). Ao indicates aorta; ccTGA, congenitally corrected transposition of the great arteries; PT, pulmonary trunk.

The anatomical disposition of the cardiac conduction system is different in the 2 forms of congenitally corrected transposition: those with the usual atrial arrangement versus those with mirror‐imaged arrangement of atrial appendages, and there are important deviations to note (Figure 1).

**Figure 2:**
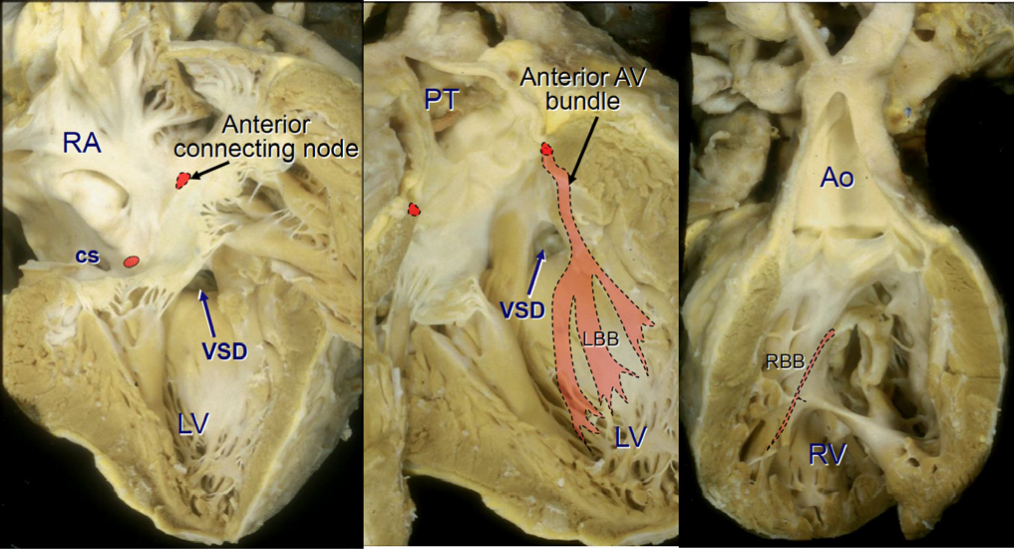
Disposition of cardiac conduction system in ccTGA. Ao indicates aorta; AV, atrioventricular; ccTGA, congenitally corrected transposition of the great arteries; cs, coronary sinus; LBB, left bundle branch; LV, left ventricle; PT, pulmonary trunk; RA, right atrium; RBB, right bundle branch; RV, right ventricle; VSD, ventricular septal defect.

Hearts with the usual atrial arrangement have a grossly abnormal disposition of the atrioventricular conduction system (Figure 1).^9^ Due to the deeply seated pulmonary outflow tract in between the ventricular septum and the mitral valve, there exists a malalignment gap between the ventricular septum and the atrial septum. The gap is filled by a large membranous septum or is occupied by a large perimembranous VSD. Consequently, a regularly situated posterior atrioventricular node is hypoplastic and cannot connect with any atrioventricular bundle. Instead, there is an anterior atrioventricular node located below the orifice of the right atrial appendage close to the lateral margin of the area of fibrous continuity between pulmonary and mitral valves. This anomalous node gives origin to the penetrating bundle of His that pierces though the fibrous trigone to continue as the atrioventricular bundle in the subendocardium of the ventricular myocardium, along the cephalad margin of the outflow tract, in the immediate subpulmonary area adjoining the pulmonary leaflets. As it reaches the ventricular septum, the bundle turns inferiorly to descend anteriorly along the septum for some length before it branches to give the left bundle branch on the right side and the right bundle branch on the left side (Figure 2). In the presence of a VSD the atrioventricular bundle is related to the defect's anterior margin, usually on the right side just off the septal crest.

By contrast, in the mirror‐imaged atrial arrangement, the sinus node is located in the left‐sided atrium in its usual position in relationship to the terminal crest and the entrance of the superior caval vein. In these hearts the atrial and ventricular septa are better aligned, allowing a regularly located atrioventricular node, at the apex of the triangle of Koch, to continue in normal fashion to an atrioventricular bundle that passes in a posteroinferior relationship to the margin of a VSD if there is one.^10^ The bundle branches descend in usual fashion with the cord‐like right bundle branch to the right side of the septum and the fan‐like left bundle branch to the left side. After giving the bundle branches, the branching bundle may continue as an anterior bundle that ends blindly, not making contact with another atrioventricular node that is situated anteriorly.^11^

There are, however, exceptions or variations to this template for hearts with the usual atrial arrangement. In cases where the pulmonary outflow tract is atretic or narrowed, septal malalignment is reduced. In these, histologic studies of a few cases have shown a sling of atrioventricular conduction system connected to both anteriorly and posteriorly situated atrioventricular nodes.^9^, ^12^ In others, although there are 2 nodes, there is no sling. The anterior node connects with an anterior bundle.^9^

Reported variations include a case with 3 atrioventricular nodes in a heart that had an accessory pathway in the left‐sided tricuspid annulus^12^ and a rare case of straddling of the mitral valve across a large perimembranous defect, which on histologic examination revealed a regularly positioned posterior node and bundle.^13^

## Cardiac Conduction Disorders in ccTGA

### Etiology

The etiology of conduction disease in ccTGA directly relates to the underlying developmental anatomical anomalies. In the absence of associated malformations, the patient may be without symptoms, remaining undiagnosed for many years and only presenting much later in life with progressive heart block, which is attributed to fibrotic changes leading to complete discontinuity of the abnormally long atrioventricular conduction bundle.^14^ Heart block may also present in infancy. The study of the conduction system by Lev and colleagues in 1963^15^ is probably the earliest to document congenitally complete atrioventricular block.

In a small series of patients, complete atrioventricular block was seen in 15 of 30 cases with normal atrial arrangement but not in any of 8 cases with associated situs inversus.^16^ Furthermore, cases with 2 AV nodes may be seen with penetrating conduction bundles that join to form a sling typically around a VSD, as first described by Monckeburg in 1913. This arrangement is classically seen in cases with the combination of atrial isomerism, an atrioventricular septal defect, and ccTGA. Perioperative AV block may occur with either physiologic or anatomic correction,^17^ and transient AV block may also be induced by cardiac catheterization.^18^

### Electrocardiographic and Electrophysiologic Features

The ECG appearance relating to ventricular activation in ccTGA is different from that seen in the structurally normal heart. As the right and left conduction bundles relate anatomically to the respective ventricles, septal activation occurs in the opposite direction, namely from right to left.^4^ This leads to a mirror image pattern in the precordial leads leading to a reversal of the normal QRS morphology, with Q waves evident in V1 and a dominant R wave but no Q wave in V6. The P‐waves and QRS axis may also vary depending on the specific atrial arrangement and location of the AV node and proximal conducting bundles,^19^ and further ECG variation may arise from other anatomical anomalies commonly seen in the condition. Manifest ventricular pre‐excitation may also be present and may mask varying degrees of intrinsic conduction disease within the AV node and proximal conducting bundles. This should be especially considered in those with a fully preexcited QRS in the absence of supraventricular tachycardia.

Conduction disease may involve either the AV node or the proximal or His bundle as well as the more distal bundle branches. In patients followed sequentially, conduction disease may progress over time, and presentation with complete AV block leading to a diagnosis of ccTGA is well recognized.^20^

An early study of the electrophysiologic properties in patients with ccTGA and conduction block including first‐degree AV block found variable sites of block occurring in the AV node, the His bundle, and the distal conduction system. In 2 patients with block distal to the His bundle, 1 presented with repeated syncope, and the other died suddenly.^7^

### Clinical Description and Natural History

Conduction disease in ccTGA may be apparent at any age from fetal life to late adulthood. From the small numbers of cases reported, the diagnosis of complete AV block in either fetal or early postnatal life does not seem to incur a higher risk of early death.^21^ A study by Huhta et al reported the annual risk of developing AV block to be 2%.^5^ In this study the major risk factor for the development of AV block was an intact ventricular septum, with AV block seen in 48% of such patients compared with 13% of those with a VSD.

The natural history of conduction disturbances in 17 patients with isolated ccTGA was reported by Daliento et al in 1986.^6^ Ten patients presented with a normal PR interval and 2 with first‐degree AV block (12%). Five patients had complete AV block (29%), but none had had it at birth, and first‐ and second‐degree AV block preceded complete AV block in 2 patients. One patient died suddenly 24 years after the onset of compete AV block, and histologic investigation disclosed fibrosis and disruption of the proximal nonbifurcating His bundle. However, it was unclear whether death was from complete heart block or an underlying arrhythmogenic myocardial substrate.^6^

The fundamental cause of morbidity and mortality in ccTGA appears to be more related to a gradual deterioration in systemic right ventricular function.^22^ The reasons for this are multifactorial in etiology, but increasingly long‐term single‐site ventricular pacing is recognized as a factor.^23^ Approximately 75% of patients with ccTGA will have other significant structural lesions including varying VSDs, pulmonary stenosis or atresia, and Ebsteinoid malformation of the left‐sided, systemic tricuspid valve, and for that reason surgery is mandated in the vast majority.^4^ Due to the decreased long‐term survival after conventional (physiologic) surgical repair, a more anatomic surgical approach has been adopted to recruit the morphologic left ventricle to the systemic circulation.^24^ Anatomic correction involves an atrial switch (Mustard or Senning procedure) coupled with the arterial switch, or a Rastelli procedure in those with a VSD and pulmonary atresia. Although no prospective comparative study will ever be performed, overall there seems to be no major benefit from an anatomic surgical approach in relation to long‐term survival of the intrinsic AV conduction system.^25^, ^26^

## Management of Conduction Disorders in ccTGA

### Follow‐Up Modalities

Approximately 10% of individuals with ccTGA present initially with complete AV block and 20% to 30% with first‐ and second‐degree AV block. The risk of surgical AV block related to anatomic repairs of ccTGA is variously reported between 3% and 16% of patients and, based on a meta‐analysis of surgical strategies in ccTGA, is higher in patients undergoing the arterial switch operation (12%) than with the Rastelli procedure. There is also a tendency of AV conduction defects to progress with age, and the incidence of complete AV block in older children and adults with ccTGA is 30% to 38%.^5^, ^27^ The risk of developing de novo AV block is ≈2%/y.^22^ For this reason, patients with ccTGA should be followed closely with annual ECG and ambulatory monitoring (even in the absence of symptoms); and cardiopulmonary stress testing can be useful (class IIa) to detect subtle deterioration in the infranodal conduction tissue that will block with faster heart rates.^28^, ^29^, ^30^

### Pacing Indications and Modalities

Permanent pacemaker implantation may be indicated in patients with congenital heart disease including ccTGA as recommended in published professional practice guidelines.^28^, ^29^, ^30^, ^31^ The decision regarding permanent pacemaker therapy must ultimately be made on an individual basis, keeping in mind the fragility of the conduction system. For example, in some ccTGA patients undergoing surgery, it may be prudent to implant prophylactic epicardial pacing leads for future use at the time of a cardiac surgical procedure even if the patient is not in imminent need of pacing, to prevent the need for another sternotomy for the sole purposes of pacing.

### Cardiac Resynchronization Therapy Indications and Modalities

Although pacemaker therapy is essential for maintaining chronotropic competence and cardiac output, reports have demonstrated an association between pacemaker implantation and the deterioration of systemic ventricular function in patients with ccTGA. More specifically, deterioration in systemic right ventricular (RV) function and worsening systemic AV valve regurgitation have been shown to be associated with subpulmonary univentricular (UniV) pacing.^23^, ^32^ Potential causes include changes in septal activation causing a septal “shift” and secondary dilatation of the systemic AV annulus with subsequent right AV valve regurgitation.^4^ Pacing‐induced ventricular dyssynchrony may also play an important role in the functional deterioration of the systemic RV in patients with ccTGA. Some studies have shown that biventricular (BiV) pacing appears to preserve systemic ventricular function in ccTGA patients. Janousek et al initially reported the benefits of cardiac resynchronization therapy (CRT) in a small case series of 8 patients with systemic RV including ccTGA, in whom there was significant improvement in the RV fractional area change (RVFAC) as well as NYHA functional class.^33^ Recently, Hofferberth et al reported their evolving experience with 53 patients with ccTGA who received primary dual‐chamber UniV pacemakers. Fourteen (26%) patients were upgraded to BiV pacemakers due to systemic ventricular dysfunction, and half of these patients demonstrated subsequent improvement in ventricular function. In the same study, 11 patients underwent primary BiV pacemaker implantation for heart block, and none developed ventricular dysfunction during short‐term follow‐up.^32^ Similarly, Yeo et al compared 22 ccTGA patients with subpulmonary UniV pacing to a relatively closely matched group of 30 unpaced ccTGA patients and 7 BiV‐paced ccTGA patients. The subpulmonary UniV‐paced patient group demonstrated progressive deterioration in the RVFAC, systemic AV valve regurgitation, and RV dilatation as well as corresponding deterioration in NYHA class compared with the unpaced patient group and the BiV‐paced patient group. The latter group showed improvement in RVFAC despite having lower baseline RVFAC.^23^ These studies give impetus for further investigation of alternative pacing modalities in ccTGA such as BiV pacing.

The response to BiV pacing is likely to be variable and may be influenced by the degree of systemic AV valve regurgitation, myocardial scarring, and type of electrical conduction delay. In a multicenter study by the Working Group for Cardiac Dysrhythmias and Electrophysiology of the Association for European Pediatric Cardiology, CRT was assessed in 109 pediatric and CHD patients including 36 patients with a systemic RV (ccTGA present in 20 patients). Reverse ventricular remodeling after CRT was significantly greater in patients with systemic left ventricle (LV) compared with those with systemic RV, and the degree of systemic AV valve regurgitation did not improve in subjects with a systemic RV after CRT.^34^ Rarely, BiV pacing may actually worsen heart failure symptoms and RVFAC. Kiesewetter et al reported 2 cases of ccTGA with subpulmonary UniV pacing, a wide QRS, and heart failure who were upgraded to CRT. After a promising early response to CRT, an adverse response with worsening heart failure and deterioration in ventricular function was encountered in both patients, leading to discontinuation of CRT.^35^ One of the explanations for the less successful reverse remodeling in patients with systemic RV may be that valvar regurgitation from a structural tricuspid valve is potentially less remediable by CRT than functional mitral regurgitation in a systemic LV. Therefore, it should be emphasized that any strategy aimed at improving RV function in ccTGA patients should ideally also include improving TV significant regurgitation.

CRT may also be considered in ccTGA patients with ventricular dysfunction and congestive heart failure without AV conduction abnormalities. However, in general, the benefits of CRT in patients with a narrow QRS are not yet established.^36^ Therefore, it is unclear whether CRT will be beneficial for ventricular remodeling in ccTGA patients with congestive heart failure without QRS prolongation.

From a procedural standpoint, unless patient size, venous obstructions, and residual lesions pose limitations, an entirely transvenous system can be implanted in ccTGA patients for the purposes of CRT. This is in contrast to CRT for patients with d‐TGA after atrial switch procedures in whom access to the systemic RV is usually obtained via a surgical procedure. In ccTGA the coronary sinus develops with the morphologic atria (ie, in the majority of cases the coronary sinus drained into the right atrium), and the venous branches, including the great cardiac vein and lateral branches, develop with the morphologic ventricles. Subsequently, the ventricular veins that drain the morphologic RV tend to be small and short, rarely draining all the way from the RV apex, which makes lead implantation challenging. However, the presence of large Thebesian veins and extensive collateralization between the RV and LV at both anterior and posterior interventricular grooves via the anterior intraventricular vein and middle cardiac vein offer alternative routes for pacing of the morphologic RV anteriorly or posteriorly.^37^ Alternatively, biV pacing may be performed by surgical placement of epicardial LV and RV pacing leads or with a hybrid approach that involves surgical placement of an epicardial RV pacing lead and transvenous placement of an endocardial LV lead.^33^, ^38^

Finally, given the relatively high percentage of ccTGA patients who will undergo either UniV or BiV pacing over their lifetimes, close patient follow‐up and meticulous monitoring of ventricular function are warranted.

### Essential Considerations for Ablation

An in‐depth understanding of the location of the AV node is vital for any supraventricular ablation in patients with any form of congenital heart disease but especially ccTGA. Ablation of typical isthmus‐dependent (cavomitral) flutter is not usually considered higher risk, and ablation can safely be performed in the standard position. Similar to ablation in hearts with normal anatomy, iatrogenic AV nodal block is less likely when the linear lesion is delivered off the septum. AV node reentry tachycardia ablation, however, requires special consideration given that the targets for ablation are the accessory atrionodal inputs—the slow pathways^39^—and the fast pathway should be avoided. In addition, if more than 1 AV nodal structure is present, multiple permutations of AV node reentry tachycardia are possible, including internodal reentry, and exact definition of the culprit circuit is crucial.

Internodal reentry utilizing a Monckeberg‐type sling whereby conduction proceeds anterograde down 1 AV node and His system and retrograde via the other can therefore be excluded by dissociating atrial and ventricular activation (confirming a lack of a 1:1 AV relationship). If, however, this arrhythmia is present, then typically 1 of the AV nodes will require ablation—but only after AV conduction is confirmed to be robust via the other AV nodal structure.

**Figure 3:**
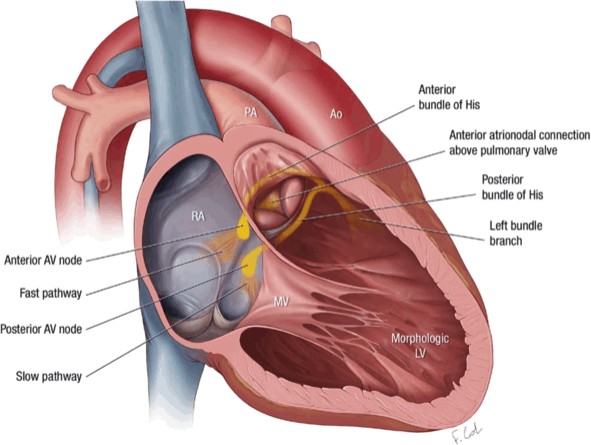
Illustration depicting the cardiac conduction system in some patients with ccTGA in whom 2 AV nodes and His bundles are present. The atrionodal connections to the posterior AV node occur via the usual slow and fast pathway locations. Atrial tissue interfaces with the anterior AV node from the usual fast pathway region but also from above the pulmonary valve along the pulmonary cusps. Ablation for elimination of AV nodal reentrant tachycardia entails defining the circuit, followed by safely targeting of the culprit slow pathway while avoiding both the fast pathway and AV nodal tissue. Ao indicates aorta; AV, atrioventricular; ccTGA, congenitally corrected transposition of the great arteries; LV, left ventricle; MV, mitral valve; PA, pulmonary artery; RA, right atrium.

As is the case in the normal heart, electrical conduction proceeding into the AV node remains silent, and the exit into atrial tissue from the AV node during tachycardia is visible as the earliest atrial activation. If other mechanisms of supraventricular tachycardia have been excluded using pacing maneuvers, and the earliest activation appears on the septum in the conventional position for the fast pathway (behind the Tendon of Todaro), then empiric, anatomically guided slow pathway ablation should be undertaken. In the situation of twin AV nodes the culprit AV node will need to be defined using resetting or subthreshold stimulation so as to identify the adjacent slow pathway to be targeted^40^, ^41^ and robust anterograde conduction via the other AV node confirmed with pacing outside of tachycardia. More commonly, anterograde conduction proceeds via the anterior AV node, and the safest empiric approach will involve ablation in the right posteroseptum in the standard location, observing closely for junctional beats and monitoring AV conduction. Unsuccessful empiric ablation here suggests that the likely culprit slow pathway is either left‐sided or involves the anterior AV nodal inputs. A stepwise approach to ablation of these slow pathways can then be considered, with the safest initial target being the left‐sided slow pathway if the operator is convinced this is not the fast pathway. The anterior AV nodal slow pathway can be targeted with ablation in the root of the pulmonary artery, pursuing atrial signals visible along the posterior aspect of the pulmonary artery cusps while also closely monitoring AV conduction (Figure 3).^39^ As a corollary to this, any ablation being undertaken near the pulmonary valve runs the risk of injury to the fragile His, which courses directly behind this structure, and the operator needs to exercise extreme caution.

Atypical exits from the AV node can also be targeted for ablation directly if these are not in the conventional fast pathway position on the septum, and the earliest atrial signal can be mapped.

## Future Directions and Conclusion

Given the “corrected” nature of the anatomy in patients with ccTGA, this disorder often presents later in life, and often with an arrhythmia heralding that an underlying congenital lesion is present. The intrinsic deficiencies in the conduction system lend themselves to premature failure, and although permanent pacing is commonly required, the optimal initial pacing strategy remains to be defined. The obvious randomized trial of univentricular versus biventricular pacing is needed for those with ccTGA, yet before we enter the realm of randomized therapies for single congenital heart disease entities, registries and larger multicenter collaborative efforts are vital and more realistically within reach. Because right ventricular systole is more of a peristaltic motion and heavily reliant on left ventricular contraction,^42^ better imaging and processing modalities are vital if we are to support the systemic right ventricle optimally through pacing. Furthermore, the insights gained will likely also advance our understanding of the arrhythmogenicity of the failing right ventricle and risk stratification/prediction against sudden cardiac death. Finally it has become impossible to imagine a future of pacing without leadless systems and more importantly without biological therapies^43^, ^44^ that can restore automaticity or conduction so that current transvenous and epicardial systems become obsolete. This latter innovation is especially attractive for those patients with ccTGA, as they frequently require pacing at a much younger age than the conventional patient with AV block^22^ and therefore have many more years exposed to the risks of intravascular leads and generator replacement surgeries.

## Sources of Funding

This work was supported by the Association for European Paediatric Cardiology (2014 Junior Scientific Research Grant), the French Society of Cardiology (2016 Bourse de Mobilité du Groupe de Rythmologie et Stimulation Cardiaque), and the Bettencourt‐Schueller Foundation (2016 Prix Jeune Chercheur) to Dr Baruteau.

## Disclosures

None.

## References

[1] Wallis GA , Debich‐Spicer D , Anderson RH . Congenitally corrected transposition. Orphanet J Rare Dis. 2011;6:22. 21569592 10.1186/1750-1172-6-22 PMC3116458

[2] van der Linde D , Konings EEM , Slager MA , Witsenburg M , Helbing WA , Takkenberg JJM , Roos‐Hesselink JW . Birth prevalence of congenital heart disease worldwide: a systematic review and meta‐analysis. J Am Coll Cardiol. 2011;58:2241–2247. 22078432 10.1016/j.jacc.2011.08.025

[3] Piacentini G , Digilio MC , Capolino R , Zorzi AD , Toscano A , Sarkozy A , D'Agostino R , Marasini M , Russo MG , Dallapiccola B , Marino B . Familial recurrence of heart defects in subjects with congenitally corrected transposition of the great arteries. Am J Med Genet A. 2005;137:176–180. 16059940 10.1002/ajmg.a.30859

[4] Warnes CA . Transposition of the great arteries. Circulation. 2006;114:2699–2709. 17159076 10.1161/CIRCULATIONAHA.105.592352

[5] Huhta JC , Maloney JD , Ritter DG , Ilstrup DM , Feldt RH . Complete atrioventricular block in patients with atrioventricular discordance. Circulation. 1983;67:1374–1377. 6851033 10.1161/01.cir.67.6.1374

[6] Daliento L , Corrado D , Buja G , John N , Nava A , Thiene G . Rhythm and conduction disturbances in isolated, congenitally corrected transposition of the great arteries. Am J Cardiol. 1986;58:314–318. 3739921 10.1016/0002-9149(86)90069-x

[7] Gillette PC , Busch U , Mullins CE , McNamara DG . Electrophysiologic studies in patients with ventricular inversion and “corrected transposition”. Circulation. 1979;60:939–945. 476896 10.1161/01.cir.60.4.939

[8] Juneja R , Rowland E , Ho SY . Atrial morphology in hearts with congenitally corrected transposition of the great arteries: implications for the interventionist. J Cardiovasc Electrophysiol. 2002;13:158–163. 11900291 10.1046/j.1540-8167.2002.00158.x

[9] Hosseinpour A‐R , McCarthy KP , Griselli M , Sethia B , Ho SY . Congenitally corrected transposition: size of the pulmonary trunk and septal malalignment. Ann Thorac Surg. 2004;77:2163–2166. 15172288 10.1016/j.athoracsur.2003.11.046

[10] Thiene G , Nava A , Rossi L . The conduction system in corrected transposition with situs inversus. Eur J Cardiol. 1977;6:57–70. 923624

[11] Wilkinson JL , Smith A , Lincoln C , Anderson RH . Conducting tissues in congenitally corrected transposition with situs inversus. Br Heart J. 1978;40:41–48. 626661 10.1136/hrt.40.1.41 PMC481972

[12] Bharati S , Rosen K , Steinfield L , Miller RA , Lev M . The anatomic substrate for preexcitation in corrected transposition. Circulation. 1980;62:831–842. 7408156 10.1161/01.cir.62.4.831

[13] Kurosawa H , Imai Y , Becker AE . Congenitally corrected transposition with normally positioned atria, straddling mitral valve, and isolated posterior atrioventricular node and bundle. J Thorac Cardiovasc Surg. 1990;99:312–313. 2299869

[14] Anderson RH , Becker AE , Arnold R , Wilkinson JL . The conducting tissues in congenitally corrected transposition. Circulation. 1974;50:911–923. 4430094 10.1161/01.cir.50.5.911

[15] Lev M , Fielding RT , Zaeske D . Mixed levocardia with ventricular inversion (corrected transposition) with complete atrioventricular block. A histopathologic study of the conduction system. Am J Cardiol. 1963;12:875–883. 14088226 10.1016/0002-9149(63)90294-7

[16] Oliver JM , Gallego P , Gonzalez AE , Sanchez‐Recalde A , Brett M , Polo L , Gutierrez‐Larraya F . Comparison of outcomes in adults with congenitally corrected transposition with situs inversus versus situs solitus. Am J Cardiol. 2012;110:1687–1691. 22935525 10.1016/j.amjcard.2012.07.039

[17] Liberman L , Silver ES , Chai PJ , Anderson BR . Incidence and characteristics of heart block after heart surgery in pediatric patients: a multicenter study. J Thorac Cardiovasc Surg. 2016;152:197–202. 27167020 10.1016/j.jtcvs.2016.03.081

[18] Mah DY , Porras D , Bergersen L , Marshall AC , Walsh EP , Triedman JK . Incidence of and risk factors for catheterization‐induced complete heart block in the pediatric cardiac catheterization laboratory. Circ Arrhythm Electrophysiol. 2014;7:127–133. 24382412 10.1161/CIRCEP.113.000731

[19] Mah K , Friedberg MK . Congenitally corrected transposition of the great arteries: situs solitus or inversus. Circ Cardiovasc Imaging. 2014;7:849–851. 25227238 10.1161/CIRCIMAGING.114.002277

[20] Kafali G , Elsharshari H , Ozer S , Celiker A , Ozme S , Demircin M . Incidence of dysrhythmias in congenitally corrected transposition of the great arteries. Turk J Pediatr. 2002;44:219–223. 12405433

[21] Sharland G , Tingay R , Jones A , Simpson J . Atrioventricular and ventriculoarterial discordance (congenitally corrected transposition of the great arteries): echocardiographic features, associations, and outcome in 34 fetuses. Heart. 2005;91:1453–1458. 15761049 10.1136/hrt.2004.052548 PMC1769184

[22] Graham TP , Bernard YD , Mellen BG , Celermajer D , Baumgartner H , Cetta F , Connolly HM , Davidson WR , Dellborg M , Foster E , Gersony WM , Gessner IH , Hurwitz RA , Kaemmerer H , Kugler JD , Murphy DJ , Noonan JA , Morris C , Perloff JK , Sanders SP , Sutherland JL . Long‐term outcome in congenitally corrected transposition of the great arteries: a multi‐institutional study. J Am Coll Cardiol. 2000;36:255–261. 10898443 10.1016/s0735-1097(00)00682-3

[23] Yeo WT , Jarman JWE , Li W , Gatzoulis MA , Wong T . Adverse impact of chronic subpulmonary left ventricular pacing on systemic right ventricular function in patients with congenitally corrected transposition of the great arteries. Int J Cardiol. 2014;171:184–191. 24374205 10.1016/j.ijcard.2013.11.128

[24] Rutledge JM , Nihill MR , Fraser CD , Smith OE , McMahon CJ , Bezold LI . Outcome of 121 patients with congenitally corrected transposition of the great arteries. Pediatr Cardiol. 2002;23:137–145. 11889523 10.1007/s00246-001-0037-8

[25] Hiramatsu T , Matsumura G , Konuma T , Yamazaki K , Kurosawa H , Imai Y . Long‐term prognosis of double‐switch operation for congenitally corrected transposition of the great arteries. Eur J Cardiothorac Surg. 2012;42:1004–1008. 22551964 10.1093/ejcts/ezs118

[26] Brizard CP , Lee A , Zannino D , Davis AM , Fricke TA , d'Udekem Y , Konstantinov IE , Brink J , Cheung MMH . Long‐term results of anatomic correction for congenitally corrected transposition of the great arteries: a 19‐year experience. J Thorac Cardiovasc Surg. 2017;154:256–265.e4. 28476422 10.1016/j.jtcvs.2017.03.072

[27] Dick M , Van Praagh R , Rudd M , Folkerth T , Castaneda AR . Electrophysiologic delineation of the specialized atrioventricular conduction system in two patients with corrected transposition of the great arteries in situs inversus (I, D, D). Circulation. 1977;55:896–900. 858185 10.1161/01.cir.55.6.896

[28] Khairy P , Van Hare GF , Balaji S , Berul CI , Cecchin F , Cohen MI , Daniels CJ , Deal BJ , Dearani JA , de Groot N , Dubin AM , Harris L , Janousek J , Kanter RJ , Karpawich PP , Perry JC , Seslar SP , Shah MJ , Silka MJ , Triedman JK , Walsh EP , Warnes CA . PACES/HRS expert consensus statement on the recognition and management of arrhythmias in adult congenital heart disease: developed in partnership between the Pediatric and Congenital Electrophysiology Society (PACES) and the Heart Rhythm Society (HRS). Endorsed by the governing bodies of PACES, HRS, the American College of Cardiology (ACC), the American Heart Association (AHA), the European Heart Rhythm Association (EHRA), the Canadian Heart Rhythm Society (CHRS), and the International Society for Adult Congenital Heart Disease (ISACHD). Heart Rhythm. 2014;11:e102–e165. 24814377 10.1016/j.hrthm.2014.05.009

[29] Baumgartner H , Bonhoeffer P , De Groot NMS , de Haan F , Deanfield JE , Galie N , Gatzoulis MA , Gohlke‐Baerwolf C , Kaemmerer H , Kilner P , Meijboom F , Mulder BJM , Oechslin E , Oliver JM , Serraf A , Szatmari A , Thaulow E , Vouhe PR , Walma E ; Task Force on the Management of Grown‐up Congenital Heart Disease of the European Society of Cardiology (ESC), Association for European Paediatric Cardiology (AEPC), ESC Committee for Practice Guidelines (CPG) . ESC guidelines for the management of grown‐up congenital heart disease (new version 2010). Eur Heart J. 2010;31:2915–2957. 20801927 10.1093/eurheartj/ehq249

[30] Brignole M , Auricchio A , Baron‐Esquivias G , Bordachar P , Boriani G , Breithardt O‐A , Cleland J , Deharo J‐C , Delgado V , Elliott PM , Gorenek B , Israel CW , Leclercq C , Linde C , Mont L , Padeletti L , Sutton R , Vardas PE ; ESC Committee for Practice Guidelines (CPG) , Zamorano JL , Achenbach S , Baumgartner H , Bax JJ , Bueno H , Dean V , Deaton C , Erol C , Fagard R , Ferrari R , Hasdai D , Hoes AW , Kirchhof P , Knuuti J , Kolh P , Lancellotti P , Linhart A , Nihoyannopoulos P , Piepoli MF , Ponikowski P , Sirnes PA , Tamargo JL , Tendera M , Torbicki A , Wijns W , Windecker S ; Document Reviewers , Kirchhof P , Blomstrom‐Lundqvist C , Badano LP , Aliyev F , Bänsch D , Baumgartner H , Bsata W , Buser P , Charron P , Daubert J‐C , Dobreanu D , Faerestrand S , Hasdai D , Hoes AW , Le Heuzey J‐Y , Mavrakis H , McDonagh T , Merino JL , Nawar MM , Nielsen JC , Pieske B , Poposka L , Ruschitzka F , Tendera M , Van Gelder IC , Wilson CM . 2013 ESC guidelines on cardiac pacing and cardiac resynchronization therapy: the Task Force on cardiac pacing and resynchronization therapy of the European Society of Cardiology (ESC). Developed in collaboration with the European Heart Rhythm Association (EHRA). Eur Heart J. 2013;34:2281–2329. 23801822 10.1093/eurheartj/eht150

[31] Epstein AE , DiMarco JP , Ellenbogen KA , Estes NAM , Freedman RA , Gettes LS , Gillinov AM , Gregoratos G , Hammill SC , Hayes DL , Hlatky MA , Newby LK , Page RL , Schoenfeld MH , Silka MJ , Stevenson LW , Sweeney MO , Tracy CM , Epstein AE , Darbar D , DiMarco JP , Dunbar SB , Estes NAM , Ferguson TB , Hammill SC , Karasik PE , Link MS , Marine JE , Schoenfeld MH , Shanker AJ , Silka MJ , Stevenson LW , Stevenson WG , Varosy PD ; American College of Cardiology Foundation, American Heart Association Task Force on Practice Guidelines, Heart Rhythm Society . 2012 ACCF/AHA/HRS focused update incorporated into the ACCF/AHA/HRS 2008 guidelines for device‐based therapy of cardiac rhythm abnormalities: a report of the American College of Cardiology Foundation/American Heart Association Task Force on Practice Guidelines and the Heart Rhythm Society. J Am Coll Cardiol. 2013;61:e6–e75. 23265327 10.1016/j.jacc.2012.11.007

[32] Hofferberth SC , Alexander ME , Mah DY , Bautista‐Hernandez V , del Nido PJ , Fynn‐Thompson F . Impact of pacing on systemic ventricular function in L‐transposition of the great arteries. J Thorac Cardiovasc Surg. 2016;151:131–138. 26410005 10.1016/j.jtcvs.2015.08.064

[33] Janousek J , Tomek V , Chaloupecký VA , Reich O , Gebauer RA , Kautzner J , Hucín B . Cardiac resynchronization therapy: a novel adjunct to the treatment and prevention of systemic right ventricular failure. J Am Coll Cardiol. 2004;44:1927–1931. 15519030 10.1016/j.jacc.2004.08.044

[34] Janousek J , Gebauer RA , Abdul‐Khaliq H , Turner M , Kornyei L , Grollmuss O , Rosenthal E , Villain E , Früh A , Paul T , Blom NA , Happonen J‐M , Bauersfeld U , Jacobsen JR , van den Heuvel F , Delhaas T , Papagiannis J , Trigo C ; Working Group for Cardiac Dysrhythmias and Electrophysiology of the Association for European Paediatric Cardiology . Cardiac resynchronisation therapy in paediatric and congenital heart disease: differential effects in various anatomical and functional substrates. Heart. 2009;95:1165–1171. 19307198 10.1136/hrt.2008.160465 PMC2699215

[35] Kiesewetter C , Michael K , Morgan J , Veldtman GR . Left ventricular dysfunction after cardiac resynchronization therapy in congenital heart disease patients with a failing systemic right ventricle. Pacing Clin Electrophysiol. 2008;31:159–162. 18233967 10.1111/j.1540-8159.2007.00963.x

[36] Ruschitzka F , Abraham WT , Singh JP , Bax JJ , Borer JS , Brugada J , Dickstein K , Ford I , Gorcsan J , Gras D , Krum H , Sogaard P , Holzmeister J ; EchoCRT Study Group . Cardiac‐resynchronization therapy in heart failure with a narrow QRS complex. N Engl J Med. 2013;369:1395–1405. 23998714 10.1056/NEJMoa1306687

[37] Bottega NA , Kapa S , Edwards WD , Connolly HM , Munger TM , Warnes CA , Asirvatham SJ . The cardiac veins in congenitally corrected transposition of the great arteries: delivery options for cardiac devices. Heart Rhythm. 2009;6:1450–1456. 19968924 10.1016/j.hrthm.2009.07.037

[38] Cecchin F , Frangini PA , Brown DW , Fynn‐Thompson F , Alexander ME , Triedman JK , Gauvreau K , Walsh EP , Berul CI . Cardiac resynchronization therapy (and multisite pacing) in pediatrics and congenital heart disease: five years experience in a single institution. J Cardiovasc Electrophysiol. 2009;20:58–65. 18775051 10.1111/j.1540-8167.2008.01274.x

[39] Noheria A , Asirvatham SJ , McLeod CJ . Unusual atrioventricular reentry tachycardia in congenitally corrected transposition of great arteries: a novel site for catheter ablation. Circ Arrhythm Electrophysiol. 2016;9:e004120 DOI: 10.1161/CIRCEP.116.004120. 27217343 10.1161/CIRCEP.116.004120

[40] Yamabe H , Misumi I , Fukushima H , Ueno K , Kimura Y , Hokamura Y . Electrophysiological delineation of the tachycardia circuit in atrioventricular nodal reentrant tachycardia. Circulation. 1999;100:621–627. 10441099 10.1161/01.cir.100.6.621

[41] Willems S , Weiss C , Shenasa M , Ventura R , Hoffmann M , Meinertz T . Optimized mapping of slow pathway ablation guided by subthreshold stimulation: a randomized prospective study in patients with recurrent atrioventricular nodal re‐entrant tachycardia. J Am Coll Cardiol. 2001;37:1645–1650. 11345379 10.1016/s0735-1097(01)01206-2

[42] Damiano RJ , La Follette P , Cox JL , Lowe JE , Santamore WP . Significant left ventricular contribution to right ventricular systolic function. Am J Physiol. 1991;261:H1514–H1524. 1951739 10.1152/ajpheart.1991.261.5.H1514

[43] Chauveau S , Anyukhovsky EP , Ben‐Ari M , Naor S , Jiang Y‐P , Danilo P , Rahim T , Burke S , Qiu X , Potapova IA , Doronin SV , Brink PR , Binah O , Cohen IS , Rosen MR . Induced pluripotent stem cell‐derived cardiomyocytes provide in vivo biological pacemaker function. Circ Arrhythm Electrophysiol. 2017;10:e004508. 28500172 10.1161/CIRCEP.116.004508 PMC5434966

[44] Maizels L , Huber I , Arbel G , Tijsen AJ , Gepstein A , Khoury A , Gepstein L . Patient‐specific drug screening using a human induced pluripotent stem cell model of catecholaminergic polymorphic ventricular tachycardia type 2. Circ Arrhythm Electrophysiol. 2017;10:e004725 DOI: 10.1161/CIRCEP.116.004725. 28630169 10.1161/CIRCEP.116.004725

